# Integrated analyses of copy number variations and gene differential expression in lung squamous-cell carcinoma

**DOI:** 10.1186/s40659-015-0038-3

**Published:** 2015-08-22

**Authors:** Zhao Yang, Bing Zhuan, Ying Yan, Simin Jiang, Tao Wang

**Affiliations:** Department of Respiratory and Critical Care Medicine, Tongji Hospital, Tongji Medical College, Huazhong University of Science and Technology, 1095 Jiefang Avenue, Wuhan, 430030 China; Department of Respiratory and Critical Care Medicine, Ningxia People’s Hospital, Yinchuan, 750011 China

**Keywords:** Lung squamous-cell carcinoma, Differentially expressed genes, Copy number variation, Copy number variation-driven genes

## Abstract

**Background:**

Although numerous efforts have been made, the pathogenesis underlying lung squamous-cell carcinoma (SCC) remains unclear. This study aimed to identify the CNV-driven genes by an integrated analysis of both the gene differential expression and copy number variation (CNV).

**Results:**

A higher burden of the CNVs was found in 10–50 kb length. The 16 CNV-driven genes mainly located in chr 1 and chr 3 were enriched in immune response [e.g. complement factor H (*CFH*) and Fc fragment of IgG, low affinity IIIa, receptor (*FCGR3A*)], starch and sucrose metabolism [e.g. amylase alpha 2A (*AMY2A*)]. Furthermore, 38 TFs were screened for the 9 CNV-driven genes and then the regulatory network was constructed, in which the GATA-binding factor 1, 2, and 3 (*GATA1*, *GATA2*, *GATA3*) jointly regulated the expression of TP63.

**Conclusions:**

The above CNV-driven genes might be potential contributors to the development of lung SCC.

## Background

Lung cancer is a leading cause of cancer mortalities worldwide [1] and the main type of lung cancer is non-small cell lung cancer (NSCLC), accounting for about 80 % of lung cancers [2]. NSCLC can be further divided into three histologic subtypes: squamous-cell carcinoma (SCC), adenocarcinoma (AC), and large-cell lung carcinoma (LCC) [3]. Of them, lung SCC represents about one third of the NSCLC burden [4]. Although numerous efforts have been made to elucidate the underlying pathogenesis and therapy of this disease over the past few decades, lung SCC is still incurable due to lack of effective therapeutic methods and late diagnosis [5, 6]. Therefore, further research in molecular pathology is still needed.

Copy number variants (CNVs) are DNA segments of ≥1 kb in length, which is present in the genome in a variable frequency [7]. Once, it was recognized that their frequency was low and associated with specific chromosome disorders directly. During the 1990s, copy number duplications and deletions were found to cause a quantity of single gene disorders [8]. Changes in DNA copy number, whether confined to specific genes or affecting whole chromosomes, have been identified as major causes of some developmental abnormalities and diseases [9]. Recently, studies related to CNVs and their roles in tumorigenesis have increased markedly [10]. For example, alteration in copy number of chromosomal regions such as 3q26.2-q29, 3p26.3-p11.1, 17p13.3-p11.2 and 9p13.3-p13.2 has been deemed as predictors of lung cancer [11]. Using molecular pathway analysis, copy number alterations in 11 genes are associated with the focal adhesion pathway in SCLC [12]. All these evidence has proven a vital role of CNV in lung cancer.

Expression profile analyses also have been performed to identify a mass of differentially expressed genes (DEGs) between normal and lung tumor samples. Wang et al. have identified 17 genes preferentially expressed in lung SCC, including four novel genes [13]. Takefumie et al. identified 40 DEGs that could separate cases with lymph-node metastasis from those without metastasis in NSCLC [14]. Furthermore, four mRNA expression subtypes including classical, basal, secretory and primitive are identified in lung LCC by gene expression profile analysis [15]. However, only few DEGs are commonly detected in different studies, which may be due to the differences in statistical methods or specimen characteristics. One possible way to increase homogeneity in these findings is an integrated analysis using both DEGs and CNVs [16, 17].

The integrated analysis of CNV and gene differential expression has previously been performed for lung AC [17], but not lung SCC. Thus, the goal of this study was to identify the CNV-driven genes in lung SCC by combining the transcriptional profile data contributed by Anders and Huber (GSE17710) [15] and CNVs data deposited in the Cancer Genome Atlas (TCGA). The identified CNV-driven genes may have a key role in elucidating the underlying mechanisms of lung SCC, thus providing a basis for developing new therapies against this disease.

## Result

### DEGs screening

A total of 428 DEGs were screened out with the cut-off value of |log_2_Ratio| > 1. Among them, 211 ones were up-regulated in lung SCC, while 217 ones were down-regulated. To explore their functions, they were subjected to GO— (p value <0.05) and KEGG (p value <0.05) pathway enrichment analyses. Noticeably, genes including matrix metallopeptidase 7 (*MMP7*), transferrin (*TF*) and serpin peptidase inhibitor clade A (*SERPINA1*) were enriched in several GO functional terms and pathways (Table 1).

**Table 1:** GO and KEGG pathway enrichment anal ysis of DEGs in lung SCC

Gene expression	Category	Term	Count	P value
Up-regulated	GOTERM_BP_FAT	GO:0006955, immune response	58	3.95E−33
	GOTERM_BP_FAT	GO:0006952, defense response	42	5.74E−20
	GOTERM_BP_FAT	GO:0009611, response to wounding	36	6.34E−17
	GOTERM_BP_FAT	GO:0006954, inflammatory response	28	1.41E−15
	GOTERM_BP_FAT	GO:0019882, antigen processing and presentation	13	2.68E−10
	GOTERM_CC_FAT	GO:0005576, extracellular region	70	8.37E−15
	GOTERM_CC_FAT	GO:0044421, extracellular region part	44	1.06E−12
	GOTERM_CC_FAT	GO:0005615, extracellular space	34	1.17E−10
	GOTERM_CC_FAT	GO:0042611, MHC protein complex	11	3.75E−09
	GOTERM_CC_FAT	GO:0042613, MHC class II protein complex	7	2.00E−06
	GOTERM_MF_FAT	GO:0008009, chemokine activity	10	2.21E−09
	GOTERM_MF_FAT	GO:0042379, chemokine receptor binding	10	3.99E−09
	GOTERM_MF_FAT	GO:0005125, cytokine activity	16	7.17E−09
	GOTERM_MF_FAT	GO:0032395, MHC class II receptor activity	6	1.97E−06
	GOTERM_MF_FAT	GO:0019865, immunoglobulin binding	4	6.21E−04
	KEGG_PATHWAY	hsa05330: allograft rejection	12	1.53E−11
	KEGG_PATHWAY	hsa05332: graft-versus-host disease	12	4.09E−11
	KEGG_PATHWAY	hsa04940: type I diabetes mellitus	12	1.00E−10
	KEGG_PATHWAY	hsa05320: autoimmune thyroid disease	12	9.74E−10
	KEGG_PATHWAY	hsa04514: cell adhesion molecules (CAMs)	17	1.01E−09
Down-regulated	GOTERM_BP_FAT	GO:0055088, lipid homeostasis	10	1.22E−08
	GOTERM_BP_FAT	GO:0042632, cholesterol homeostasis	9	1.87E−08
	GOTERM_BP_FAT	GO:0055092, sterol homeostasis	9	1.87E−08
	GOTERM_BP_FAT	GO:0065005, protein-lipid complex assembly	6	2.18E−07
	GOTERM_BP_FAT	GO:0034377, plasma lipoprotein particle assembly	6	2.18E−07
	GOTERM_CC_FAT	GO:0005615, extracellular space	35	4.43E−12
	GOTERM_CC_FAT	GO:0044421, extracellular region part	39	1.70E−10
	GOTERM_CC_FAT	GO:0005576, extracellular region	52	3.66E−07
	GOTERM_CC_FAT	GO:0034385, triglyceride-rich lipoprotein particle	6	4.09E−06
	GOTERM_CC_FAT	GO:0034361, very-low-density lipoprotein particle	6	4.09E−06
	GOTERM_MF_FAT	GO:0008289, lipid binding	19	6.35E−06
	GOTERM_MF_FAT	GO:0004866, endopeptidase inhibitor activity	11	8.62E−06
	GOTERM_MF_FAT	GO:0005496, steroid binding	8	1.02E−05
	GOTERM_MF_FAT	GO:0004867, serine-type endopeptidase inhibitor activity	9	1.31E−05
	GOTERM_MF_FAT	GO:0030414, peptidase inhibitor activity	11	1.38E−05
	KEGG_PATHWAY	hsa04610: complement and coagulation cascades	7	3.32E−04
	KEGG_PATHWAY	hsa00830: retinol metabolism	6	8.02E−04
	KEGG_PATHWAY	hsa00860: porphyrin and chlorophyll metabolism	5	9.97E−04
	KEGG_PATHWAY	hsa00053: ascorbate and aldarate metabolism	4	0.0015
	KEGG_PATHWAY	hsa00040: pentose and glucuronate interconversions	4	0.0018

*GO* gene ontology, *KEGG* kyoto encyclopedia of genes and genomes, *DEGs* differentially expressed genes, *SCC* squamous-cell carcinoma, *BP* biological process, *CC* cellular component, *MF* molecular function

### Identification of CNV-driven DEGs

**Fig. 1:**
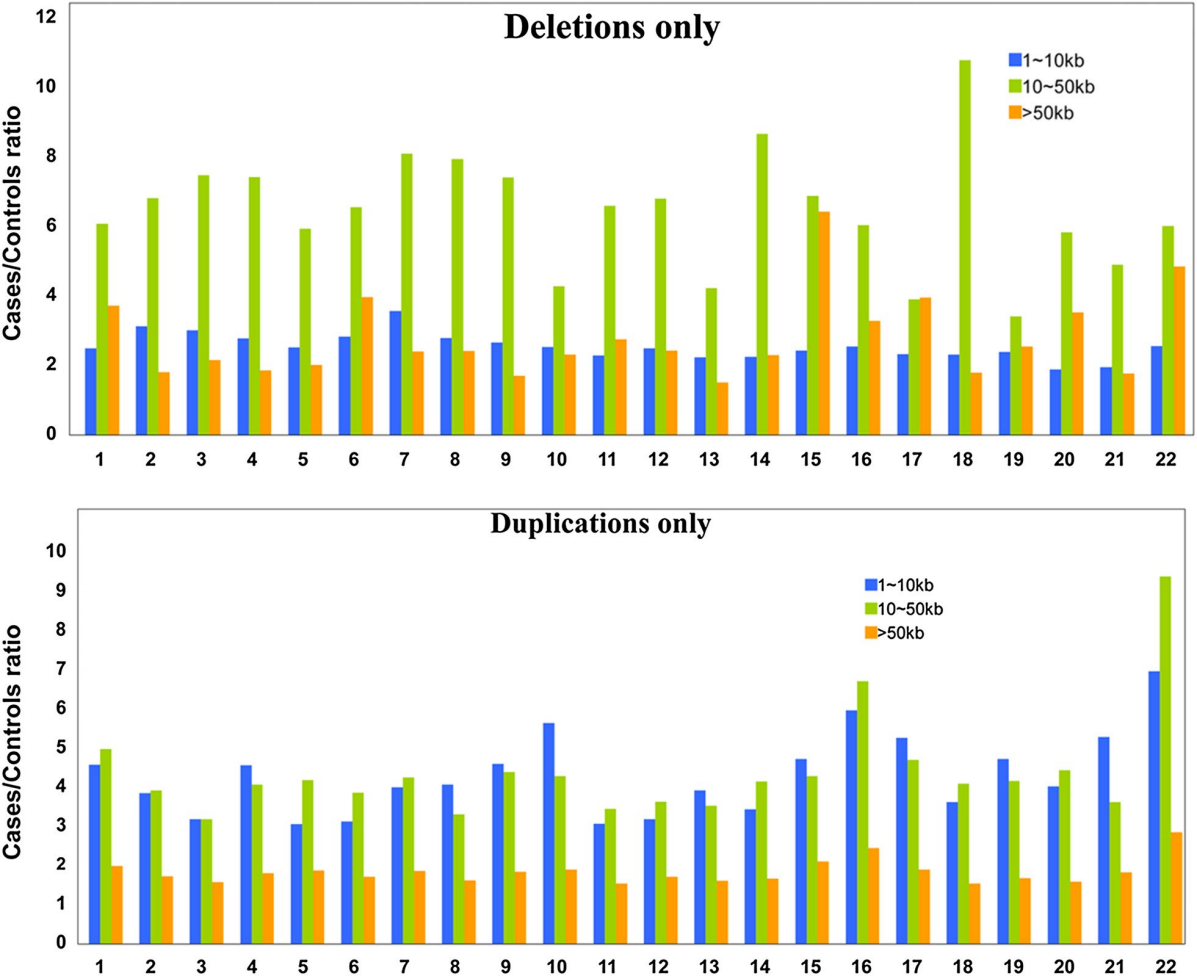
The ratios of the number of copy number deletions/duplications in cases to that in controls at different lengths

It is found that regions of 1–10 kb long had the most copy number deletions (Table 2), while regions of >50 kb long had the most copy number duplications (Table 3). Overall, the ratio of copy number deletions in tumors to those in controls was larger in regions of 10–50 kb long, mostly larger than 6, and the ratio of copy number duplications in tumors to those in controls was lowest in regions of >50 kb long (Fig. 1).

**Table 2:** Distribution of copy number deleti ons on chromosomes

Chr	Deletions only								
	Observed CNV in cases and controls	Ratio of case/control	p values	Observed CNV in cases and controls	Ratio of case/control	p values	Observed CNV in cases and controls	Ratio of case/control	p values
	1–10 kb			10–50 kb			>50 kb		
1	1709	2.49539	0.8626	384	6.08127	0.3246	170	3.72008	0.2772
2	1642	3.13118	1.00E−04	509	6.81826	0.0332	237	1.80857	0.0413
3	1611	3.01258	0.7392	500	7.48133	0.9189	266	2.15987	0.8298
4	1904	2.77845	0.8719	520	7.42289	0.9481	251	1.86097	0.3325
5	1483	2.52167	0.0617	543	5.93514	0.0453	249	2.01002	0.6479
6	1440	2.82868	0.2306	362	6.5556	0.0581	118	3.96903	0.5206
7	1248	3.57254	0.7114	368	8.09861	0.6195	113	2.40595	0.8872
8	1388	2.79406	0.0007	371	7.95181	0.882	306	2.41128	0.4959
9	1195	2.66351	1.00E−04	391	7.41424	0.0011	495	1.69698	0.7884
10	1107	2.5371	0.2834	269	4.27999	0.5673	208	2.3065	0.0204
11	1183	2.28072	0.0046	282	6.60225	0.0169	138	2.7495	0.0148
12	893	2.49211	0.0323	152	6.80094	0.0364	35	2.42966	0.0054
13	932	2.23366	0.3331	272	4.2262	0.0271	94	1.51505	0.5151
14	741	2.25316	0.0106	221	8.66705	0.2036	118	2.3046	0.6436
15	623	2.4265	0.06	157	6.87918	0.0042	139	6.42084	0.0004
16	647	2.54643	0.5412	216	6.04562	0.0075	122	3.28046	0.1362
17	594	2.33232	0.2435	121	3.90574	0.3034	72	3.95823	0.0278
18	698	2.30858	0.9919	135	10.792	0.7888	74	1.79206	0.2417
19	405	2.39362	0.9983	121	3.42131	0.9996	44	2.54418	0.7119
20	427	1.88614	0.0405	78	5.83578	0.4637	24	3.52891	0.1368
21	345	1.94557	0.4154	85	4.8969	0.0999	82	1.77643	0.025
22	319	2.5579	0.0145	81	6.01787	0.0148	44	4.84881	0.0547

**Table 3:** Distribution of copy number duplications in chromosomes

Chr	Duplications only								
	Observed CNV in cases and controls	RATIO of Case/Control	p values	Observed CNV in cases and controls	Ratio of Case/Control	p values	Observed CNV in cases and controls	Ratio of Case/Control	p values
	1 ~ 10 kb			10 ~ 50 kb			>50 kb		
1	1924	4.56541	1.00E−04	2621	4.97426	1.00E−04	11,911	1.98385	0.0022
2	1912	3.84581	0.0086	2627	3.91763	1.00E−04	13,136	1.71774	1.00E−04
3	2070	3.17944	0.0012	2669	3.17624	1.00E−04	13,120	1.56874	1.00E−04
4	1724	4.56274	0.8782	2410	4.06176	0.2453	11,164	1.80262	0.999
5	1272	3.06097	1	1866	4.18235	0.9991	9063	1.87245	0.9003
6	1560	3.12497	0.0007	2049	3.86111	1.00E−04	9809	1.71417	0.0002
7	1782	3.99707	0.9365	2523	4.25108	0.1637	9972	1.86011	0.9153
8	1667	4.06267	1.00E−04	2150	3.30777	1.00E−04	10,659	1.61986	1.00E−04
9	1178	4.59415	0.0016	1552	4.38777	0.1466	7928	1.83387	0.0005
10	986	5.63493	0.0195	1243	4.28617	0.0254	7138	1.89335	1.00E−04
11	1167	3.06759	1.00E−04	1598	3.44469	1.00E−04	8478	1.52969	1.00E−04
12	1254	3.18495	0.3166	1432	3.62956	0.0677	7431	1.70661	0.0044
13	700	3.91295	0.1717	1011	3.52657	0.0039	5535	1.60301	0.0112
14	655	3.43045	0.342	1070	4.14655	0.5122	5294	1.66644	0.0106
15	574	4.72049	0.3773	930	4.28337	1.00E−04	4371	2.0948	1.00E−04
16	726	5.96031	0.0747	1094	6.71092	0.1114	5387	2.4445	1.00E−04
17	597	5.26255	0.0105	995	4.69484	1.00E−04	5477	1.89667	0.0003
18	719	3.61849	1	849	4.09121	1	4894	1.54215	1
19	520	4.72055	0.8769	943	4.15877	0.9467	5166	1.67776	1
20	594	4.01529	0.0188	728	4.42725	0.0907	3890	1.58039	0.0172
21	340	5.28483	0.9071	558	3.61798	0.8819	2724	1.8263	0.2397
22	526	6.95908	0.0003	689	9.38731	0.0032	2976	2.84862	1.00E−04

**Fig. 2:**
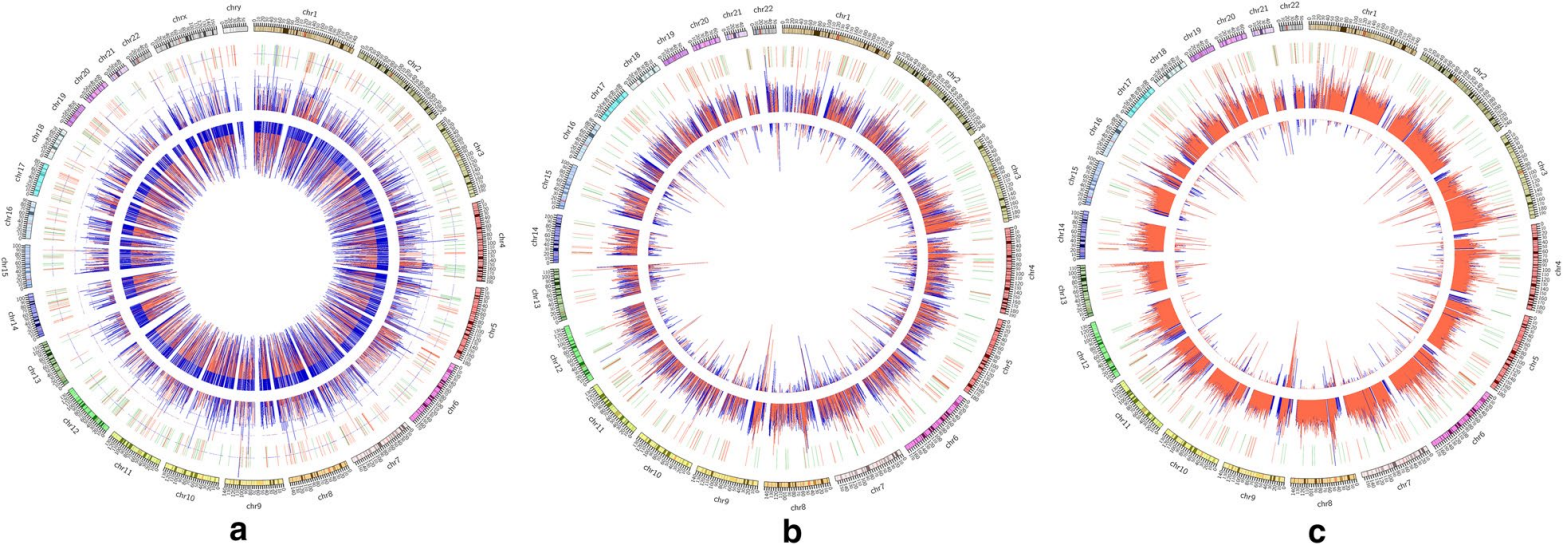
Genomic distributions of differentially expressed genes (DEGs) and copy number variations (CNV) related to lung squamous-cell carcinoma using Circos-plots. **a**, **b** and **c** Represent the genomic distribution of CNV regions of 1–10 kb, 10–50 kb and >50 kb, respectively. The outermost *bars* in a *circle labeled with numbers* represent chromosomes; the second outermost *circle* represents DEGs (*red* and *green* indicating up-regulated and down-regulated DEGs, respectively); the first innermost circle (*inward*) represent copy number deletions, and the second innermost circle (*outward*) representing copy number duplications (a *red line* indicates a CNV occurring in controls, a blue line indicates a CNV occurring in cases, with the length of the line determined by the copy number)

A total of 313 CNV genes related to lung SCC were detected, which were only found in more than 80 % cases, while not in the controls. Then these CNVs were checked for overlap with the DEGs. A total of 163 overlapping CNV genes that were also detected by microarrays were obtained, of which 24 genes displayed significantly different expression. Furthermore, 16 CNV-driven genes were identified with the same expressional tendencies, namely copy number increasing with the expression level (Table 4). Among them, seven (*FCGR3A*, *FCGR2B*, *AMY2A*, *AMY2B*, *CFH*, *LCE1D*, and *CFHR3*) were located on chr 1, three on chr 3 (*TP63*, *MUC4*, and *MUC20*), and one on chr 4, 5, 6, 7, 16 and 19, respectively (Fig. 2).

**Table 4:** CNV-driven genes in lung SCC

Chr	Gene	log2(copy number)	log2(FC)	Karyotype
1	FCGR3A	1.13334	3.91384	q23.3
1	FCGR2B	1.14834	2.52075	q23.3
1	AMY2A	1.06865	2.50174	p21.1
1	AMY2B	1.58959	1.94116	p21.1
1	CFH	1.06624	1.47934	q31.3
1	LCE1D	1.23411	1.41679	q21.3
1	CFHR3	1.20817	1.35526	q31.3
3	TP63	1.04434	3.18336	q28
3	MUC4	1.35013	2.38177	q29
3	MUC20	1.63525	2.25619	q29
4	TMPRSS11E	1.11569	1.78099	p16.3
5	ZDHHC11	1.07998	1.03782	q14.1
6	HLA-DQA1	1.12618	1.73272	p21.32
7	ARHGEF5	1.07093	1.46641	q35
16	CES1	1.68967	2.06824	q12.2
19	LILRB5	1.08841	1.39769	q13.42

*CNV* copy number variation, *FC* fold change

### GO and pathway analysis of CNV-driven genes

To functionally understand the CNV-driven genes, GO (p value <0.05) and KEGG pathway (p value <0.05) enrichment analyses were also performed. According to GO annotation, the CNV-driven genes were mainly functionally related to the biological process term immune response (such as *CFH*, *FCGR3A*), and cellular component term amylase activity (*AMY2A* and *AMY2B*), as well as molecular function terms amylase activity and IgG binding. Meanwhile, the CNV-driven genes were significantly enriched in pathways of systemic lupus erythematosus, starch and sucrose metabolism pathway (amylase alpha 2A, *AMY2A*) (Table 5).

**Table 5:** GO and KEGG pathway enrichment analysis of CNV-driven genes

Category	Term	P value	Genes
GOTERM_BP_FAT	GO:0006955, immune response	0.002390231	*FCGR2B*, *LILRB5*, *CFH*, *FCGR3A*, *HLA-DQA1*
GOTERM_CC_FAT	GO:0005576, extracellular region	0.001678775	*CFHR3*, *TMPRSS11E*, *MUC20*
			*CFH*, *AMY2B*, *FCGR3A*, *AMY2A*, *MUC4*
GOTERM_MF_FAT	GO:0016160, amylase activity	0.002309112	*AMY2B*, *AMY2A*
GOTERM_MF_FAT	GO:0004556, alpha-amylase activity	0.002309112	*AMY2B*, *AMY2A*
GOTERM_MF_FAT	GO:0019864, IgG binding	0.006146971	*FCGR2B*, *FCGR3A*
KEGG_PATHWAY	hsa05322: systemic lupus erythematosus	0.00534888	*FCGR2B*, *FCGR3A*,* HLA-DQA1*
KEGG_PATHWAY	hsa00500: starch and sucrose metabolism	0.048568802	*AMY2B*, *AMY2A*

## Discussion

In the present study, using both transcriptional profile data and CNV data, we identified genes with differential expression that may be caused by CNV. DEGs such as *MMP7*, *TF* and *SERPINA1* might be associated with lung SCC. The up-regulated *MMP7* was enriched in several significant GO functional terms such as negative regulation of cellular protein metabolic process, male gonad development and sterol homeostasis in our study. *MMP7* belongs to metalloproteinase (*MMP*) family and plays a role in the breakdown of extracellular matrix [18]. It is shown that the polymorphism in *MMP7* promoter increases susceptibility to esophageal SCC [19]. Moreover, down-regulated TF enriched in significant GO functional terms including extracellular space and extracellular region part. It functioned as an iron transporter [20]. The receptor of *TF* contributes to NSCLCs and it may be an indicator of poorer prognosis in certain groups of patients [21]. Up-regulated *SERPINA1* was enriched in several significant GO function terms such as extracellular region and extracellular region part as well. It is a serine protease inhibitor and contributes to chronic obstructive pulmonary disease [22]. There is evidence that *SERPINA1* is a biomarker for progression of cutaneous SCC [23]. All these evidences suggest that *MMP7*, *TF* and *SERPINA1* may play pivotal roles in lung SCC.

CNV analysis presents a higher burden of the CNVs in length of 10–50 kb in lung SCC. A significant increase in CNV burden was observed in most of the individual chromosomes. It is reported that the increased burden of structural variation is a genetic risk factor for cancer [24]. Hence, no wonder the structural variation in length is correlated with lung SCC. Our study provides a hint for the etiology of lung SCC, which is the fragile or disordered genomes, specifically due to the structural variations of copy number in length of 10–50 kb.

Among the 16 overlapping genes, 7 were located on chr 1, and 3 on chr 3, indicating CNV related to lung SCC may mainly occur on chromsomes 1 and 3. According to GO functional analysis and KEGG pathway annotation, these 16 genes may be mainly involved in lung SCC by influencing starch and sucrose metabolism (e.g. *AMY2A*, *AMY2B*), and the immune response (e.g. *FCGR3A*, *FCGR2B*, *CFH*, *HLA*-*DQA1*). *AMY2A* and *AMY2B* encoding amylases that hydrolyze 1,4-α-glucoside bonds in oligosaccharides and polysaccharides, thus is necessary for the digestion of dietary starch and glycogen [25]. Kang et al. have reported that *AMY2A* is a possible tumor-suppressor gene of 1p21.1 in gastric carcinoma [26]. Furthermore, Xi et al. have detected CNV of *AMY2A* using the Bayesian information criterion [27]. Thus, it may be inferred alteration in *AMY2B* and *AMY2A* copy numbers may have a role in lung SCC occurrence via the starch and sucrose metabolism pathway. *FCGR3A* encoding a cell surface molecule *CD16a*, a member *CD16* gene family, which is much similar to *FCGR3B* on chromosome 1. Zhou et al. have reported that it is CNV of *FCGR3A* other than *FCGR3B* and *FCGR2B* that is involved in anti-GBM disease [28]. Meanwhile, a recent study also discovered a role of *CD16* signaling receptor in antibody-dependent cancer cell killing [29]. Thus, it may be speculated that alterations in the copy number of these two genes might influence the immune processes, further contributing to lung SCC. However, CNV of the two genes have never been reported in lung SCC. More significantly, *CFH* at chr 1 is a member of the regulator of complement activation gene cluster. It was found that *CFH* sensitizes NSCLC cells to complement attack and inhibit the growth of tumor cells [30]. Therefore, the presence of CNV located in chr 1 and chr 3 might be potential contributory factors to the development of lung SCC.

TP63 (transformation-related protein 63) encoded by TP63 gene, along with p53 and p73, constitutes the p53 gene family of transcription factors [31]. Noticeably, TAp63 may functionally similar to p53 as it is reported to transactivate multiple p53 downstream targets. However, p53 has only one promoter with three conserved domains, whereas either p63 or p73 has two promoters, thus each having two isotypes: one containing TA domain (TAp63, TAp73), and the other containing no TA domain (ΔNp63 and ΔNp73). Wu et al. investigated the cell functions by introducing TAp63α and ΔNp63α into Saos2 cells using adenovirus expression vectors and subsequently detecting the gene profiles using DNA microarrays, and they have found that p63 can regulate a wide range of various cellular functions, such as cell cycle control, stress, and signal transduction, which are critical events in cancer and development [32]. It thus may be inferred that CNV of TP63 might have a role in lung SCC by altering the expression of its downstream genes, although no copy number variation has been reported in p63 gene so far.

## Conclusions

In this study we conducted an integrated analysis of transcriptional profile and CNV of lung SCC, and finally screened 16 CNV-driven genes. The variation in these gene copy number is speculated to have a role in lung SCC occurrence. For example, *FCGR3A*, *FCGR2B* and *HLA*-*DQA1* may function via the systemic lupus erythematosus pathway, and *AMY2B* and *AMY2A* may participate in lung SCC via starch and sucrose metabolism pathway. Our work provides new insights into the mechanisms underlying lung SCC, and also suggest some new targets for therapy of lung SCC. However, their roles in lung SCC require further experimental validation. The genes *AMY2A*, *CFH*, and *TP63* However, more in-depth studies are needed in order to verify our findings.

## Methods

As the paper did not involve any human or animal study, ethical approval was not required.

### Source of gene expression data and CNV data

Transcriptional profile of GSE17710 [33] was downloaded from Gene Expression Omnibus (GEO) database (http://www.ncbi.nlm.nih.gov/geo/), which was annotated using the platform of GPL9053 (Agilent-UNC-custom-4X44k). This dataset was collected from both the tumor tissues and adjacent normal tissues of 56 lung SCC patients. CNV data were downloaded from the TCGA database (https://tcga-data.nci.nih.gov/tcga/dataAccessMatrix.htm?mode=ApplyFilter&showMatrix=true&diseaseType=LUSC&tumorNormal=TN&tumorNormal=T&tumorNormal=NT&platformType=1&platformType=4&platformType=40) in Dec., 2013. Only data of level 3 were accessible and downloaded, which included CNV information (segment mean value) in both the tumor issues and matched adjacent normal tissues (control) from 513 patients with lung SCC using Affymetrix Genome-Wide Human SNP 6.0 array containing 1.8 million SNP and CNV probes. A segment mean value is log2 transformed ratio of the detected copy number in either the tumor or normal tissues to the copy number 2 that is detected in a normal human using hg19 as reference genome. A value larger than one indicates a copy number increase, and a value smaller than one means a copy number depletion.

### Preprocessing of transcriptional profile data

The probe-level data of transcriptional profile were first converted into expression measures. DEGs were identified between the tumor tissues and adjacent normal tissues with the cut-off criteria of |log_2_FC (Fold change)| > 1.

#### Preprocessing of CNV microarray data

First, the distribution of CNVs (copy number deletion or increase) on the 22 chromosomes was investigated in both the cases and controls, and the number of CNV regions of 1–10 kb, 10–50 kb and >50 kb in length on each chromosome was calculated, respectively. Permutation test was performed to calculate the *P* value of CNVs in either the cases or controls in each chromosome based on 1000 replicates. Next, Circos software was used to display the distribution of on each chromosome in both tumors and controls.

### Identification of lung SCC-related CNVs

First, genes located within CNV regions were identified according to the human hg19 reference genome, and its copy vari in an identified gene was also calculated. Next, a gene that is related to lung SCC with CNV was retained only when its CNV was not detected in controls but detected in more than 80 % ceases. The segment mean value and the copy number were denoted as 0 and 1, respectively when missing in a sample.

### Identification of CNV-driven genes

The association between the copy number difference data and differential expression data was analyzed to screen CNV-driven genes. A CNV-driven gene was defined only when the differential expression trend was consistent with the copy number change, namely an up-regulated gene should also have an increased copy number; vice versa.

### Functional annotation and pathway enrichment analysis of CNVs

The functional enrichment analysis of the DEGs and CNV-driven genes was carried out using Database for Annotation, Visualization and Integrated Discovery (DAVID) software based on the gene ontology (GO) and kyoto encyclopedia of genes and genomes (KEGG) pathway databases [34]. P < 0.05 was set as the cut-off.
